# SARS-CoV-2 501Y.V2 (B.1.351) Elicits Cross-Reactive Neutralizing Antibodies

**DOI:** 10.1056/NEJMc2104192

**Published:** 2021-04-07

**Authors:** Thandeka Moyo-Gwete, Mashudu Madzivhandila, Penny L. Moore, Complete List of Authors, for Publication at NEJM.ORG:, Thandeka Moyo-Gwete, Mashudu Madzivhandila, Zanele Makhado, Frances Ayres, Donald Mhlanga, Brent Oosthuysen, Bronwen E. Lambson, Prudence Kgagudi, Houriiyah Tegally, Arash Iranzadeh, Deelan Doolabh, Lynn Tyers, Lionel R. Chinhoyi, Mathilda Mennen, Sango Skelem, Gert Marais, Constantinos K. Wibmer, Jinal N. Bhiman, Veronica Ueckermann, Theresa Rossouw, Michael Boswell, Tulio de Oliveira, Carolyn Williamson, Wendy A. Burgers, Ntobeko Ntusi, Lynn Morris

**Affiliations:** National Institute for Communicable Diseases Johannesburg, South Africa; KwaZulu-Natal Research Innovation and Sequencing Platform (KRISP) Durban, South Africa; University of Cape Town Cape Town, South Africa; National Institute for Communicable Diseases Johannesburg, South Africa; Steve Biko Academic Hospital Pretoria, South Africa; University of Pretoria Pretoria, South Africa; Steve Biko Academic Hospital Pretoria, South Africa; KwaZulu-Natal Research Innovation and Sequencing Platform (KRISP) Durban, South Africa; University of Cape Town Cape Town, South Africa

**To the Editor:** The 501Y.V2 lineage (B.1.351), identified in South Africa in October 2020^1^ contains mutations that confer increased resistance to convalescent and vaccinee plasma and some monoclonal antibodies^2,3,4^. However, the immune response to 501Y.V2 is unknown. Similarly, the ability of antibodies elicited by 501Y.V2 infection to cross-react with other variants is unknown, but has implications for the ability of second-generation vaccines, based on the 501Y.V2 spike, to protect against infection by the original and emerging SARS-CoV-2 lineages^5^.

We characterized a cohort of COVID-19 patients hospitalized in the Groote Schuur Hospital, Cape Town (Table S1), after the emergence and dominance of 501Y.V2 in South Africa. Blood samples from 89 patients were collected between 31 December 2020 and 15 January 2021, and sequences from 28/89 (31%) randomly selected patients were all shown to be 501Y.V2 by phylogenetic analysis (Figure S1A). Furthermore, at this time, the epidemic in Cape Town, and South Africa, was dominated by 501Y.V2, accounting for over 90% of infections (Fig. S1B). No patient reported prior SARS-CoV-2 infection.

We first assessed the binding and neutralizing antibody responses of these patients to the 501Y.V2 spike protein. As with the original variant, 501Y.V2 elicits high titer binding and neutralizing antibody responses (Fig. S2). Furthermore, binding antibodies to the receptor binding domain (including the whole subdomain 1) and full spike protein of the original and 501Y.V2 variants were highly correlated. Titration of a subset of 46 samples revealed that plasma samples had higher titers to the spike of 501Y.V2 than to the original variant (average reduction of 1.7-fold), but high-level binding to the original variant remained (Fig. S3).

We previously reported substantially lower neutralization of the 501Y.V2 variant by plasma from individuals infected with the original variant (Fig. 1A, Figure S4A)^2^. Here, we performed the reverse experiment by assessing the cross-reactivity of the plasma neutralizing responses in the Groote Schuur Hospital 501Y.V2 cohort against the original variant and 501Y.V3 (P.1), the variant first described in Brazil. We first tested 57 Groote Schuur Hospital patient sera against both 501Y.V2 and the original variant (Fig. 1B). Strikingly, 53/57 plasma samples maintained neutralization activity against the original variant, with an average fold reduction of 3 (Fig. 1B) and a geometric mean titer (GMT) of 203 (95% confidence interval:141-292) (Figure S5A). When we limited the analysis to 22/28 donors for whom sequencing data proved infection with 501Y.V2, and who had antibody binding titers, we observed the same pattern (Fig. 1C). Lastly, we tested a subset of 10 plasma samples against the 501Y.V3 (P.1) variant. We showed high levels of neutralization of this variant, with some samples showing increased potency, a finding that may be due to the very different N-terminal domain regions of these variants (Fig. 1D)

Overall, we show that 501Y.V2 elicits robust neutralizing antibodies that neutralize both the original variant and 501Y.V3 (P.1), indicating high levels of cross-reactivity. Our data indicate that vaccines built upon the spike protein of 501Y.V2 may be promising candidates for the elicitation of cross-reactive neutralizing antibodies to SARS-CoV-2.

## Supplementary Material

Supplement

## Figures and Tables

**Figure 1:**
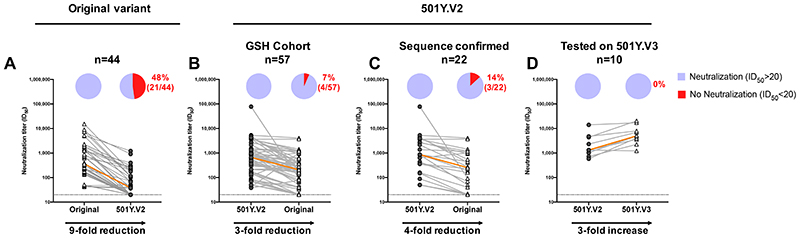
Neutralizing antibodies elicited by 501Y.V2 infection are more cross-reactive than those from patients infected with the original variant. (A) Plasma samples from patients infected with the original variant and (B-C) 501Y.V2-infected GSH cohort samples were compared for their neutralization cross-reactivity against other variants (n=57). In (C), the analysis was limited to those samples where sequencing confirmed infection by 501Y.V2 (n=22). (D) A subset of samples (n=10) was assayed against 501Y.V3 pseudoviruses. The orange line indicates the slope between the median neutralization potency of the samples tested. In the pie charts, purple indicates the proportion of samples with neutralization activity and red the proportion of samples with no detectable neutralization activity. The threshold of detection for the neutralization assay is ID50>20. All experiments were performed in duplicate and the average value shown. Data for the original variant plasma was taken from Wibmer et al., 2021, Nature Medicine.
