# Supplementary material for: SARS-CoV-2 501Y.V2 (B.1.351) Elicits Cross-Reactive Neutralizing Antibodies
Source: N Engl J Med. Author manuscript; Available in PMC 2021 Jul 23. (PMC8063886; doi:10.1056/NEJMc2104192)
Supplement: Supplement [file EMS126603-supplement-Supplement.pdf]

Supplemental Appendix

Table of Contents

List of investigators 2
Cohort Description and Methods 3
Acknowledgements 12
Author Contributions 12
Conflicts of Interest 13
References 13

List of investigators

Thandeka Moyo-Gwete*[1,2], Mashudu Madzivhandila*[1], Zanele Makhado[1], Frances Ayres[1], Donald Mhlanga[1], Brent Oosthuysen[1], Bronwen E. Lambson[1,2], Prudence Kgagudi[1], Houriiyah Tegally[3], Arash Iranzadeh[4], Deelan Doolabh[4], Lynn Tyers[4] Lionel R. Chinhoyi[5,6,7], Mathilda Mennen[5,6,7], Sango Skelem[5,6,7], Gert Marais[4,8], Constantinos Kurt Wibmer[1], Jinal N Bhiman[1,9], Veronica Ueckermann[10], Theresa Rossouw[11], Michael Boswell[10], Tulio de Oliveira[3,12,13], Carolyn Williamson[4,5,8], Wendy A Burgers[4,5], Ntobeko Ntusi[5,6,7], Lynn Morris[1,2,12], Penny L Moore[+1,2,12]

[1]National Institute for Communicable Diseases of the National Health Laboratory Service, Johannesburg, South Africa. [2]Antibody Immunity Research Unit, School of Pathology, Faculty of Health Sciences, University of the Witwatersrand, Johannesburg, South Africa. [3]KwaZulu-Natal Research Innovation and Sequencing Platform (KRISP), Department of Laboratory Medicine & Medical Sciences, University of KwaZulu-Natal, Durban, South Africa. [4]Institute of Infectious Disease and Molecular Medicine, Division of Medical Virology, Department of Pathology, University of Cape Town, Cape Town, South Africa. [5]Wellcome Centre for Infectious Diseases Research in Africa, University of Cape Town, Cape Town, South Africa. [6]Division of Cardiology, Department of Medicine, University of Cape Town and Groote Schuur Hospital, Cape Town, South Africa. [7]Hatter Institute for Cardiovascular Research in Africa, Faculty of Health Sciences, University of Cape Town, Cape Town, South Africa. [8]National Health Laboratory Services, Groote Schuur Hospital, Cape Town, South Africa. [9]Department of Virology, School of Pathology, Faculty of Health Sciences, University of the Witwatersrand, Johannesburg, South Africa. [10]Division for Infectious Diseases, Department of Internal Medicine, Steve Biko Academic Hospital and University of Pretoria, Pretoria, South Africa. [11]Department of Immunology, Faculty of Health Sciences, University of Pretoria, Pretoria, South Africa. [12]Centre for the AIDS Programme of Research in South Africa (CAPRISA), Durban, South Africa. [13]Department of Global Health, University of Washington, Seattle, USA.

*these authors contributed equally to this article
[+]corresponding author: Penny L Moore (pennym@nicd.ac.za)

Cohort Description and Methods

Cohort

Plasma samples were obtained from hospitalized COVID-19 patients (n=89) with moderate disease admitted to Groote Schuur Hospital cohort, Cape Town from 30 December 2020 – 15 January 2021 during the second wave in South Africa. All patients were aged ≥18 years and were HIV negative. This study received ethics approval from the Human Research Ethics Committee of the Faculty of Health Sciences, University of Cape Town (R021/2020). All patients had polymerase chain reaction (PCR) confirmed SARS-CoV-2 infection a median of 7 days (IQR 3-11) before blood collection. Clinical folders were consulted for 87/89 participants and none showed evidence of prior symptomatic COVID-19 disease.

We also used data from a previously described cohort infected with the original D614G variant during the first wave, for comparison[1]. The Pretoria COVID study cohort consisted of adults (age > 18 years) who had PCR-confirmed SARS-CoV-2 infections, with moderate to severe COVID-19, and were admitted to Steve Biko Academic Hospital (Pretoria, South Africa) from April to September 2020. Blood samples were collected at admission and a second follow-up visit, or at discharge from hospital (whichever was sooner). Admission occurred at a median of 3 days (IQR:2-5) after PCR testing, and follow-up samples were taken a median of 10 days (IQR:9-13) after testing. Ethics approval was received from the University of Pretoria, Human Research Ethics Committee (Medical) (247/2020).

SARS-CoV-2 spike genome sequencing

Sequencing of the spike was performed using swabs obtained from 28 randomly collected Groote Schuur Hospital patients. Swabs were stored as follows: from mid-December 2020 onwards, all SARS-CoV-2 positive swabs collected at the Groote Schuur Hospital from the wards or testing center were stored. For patients at Groote Schuur who had swabs taken elsewhere, a maximum of 500 swabs per week were stored. If the total number of swabs exceeded 500 per week, a regional random proportional selection algorithm was applied due to storage restrictions. A CT cutoff of less than 30 was used as these would likely be successfully sequenced. Swabs from the other participants were not stored. RNA sequencing was performed as previously published[2]. Briefly, RNA extracted participants was used to synthesize cDNA using the Superscript IV First Strand synthesis system (Life Technologies, Carlsbad, CA) and random hexamer primers. SARS-CoV-2 whole genome amplification was performed by multiplex PCR using primers designed on Primal Scheme (http://primal.zibraproject.org/) to generate 400 bp amplicons with a 70 bp overlap covering

the SARS-CoV-2 genome. PCR products were cleaned up using AMPure XP magnetic beads (Beckman Coulter, CA) and quantified using the Qubit dsDNA High Sensitivity assay on the Qubit 3.0 instrument (Life Technologies Carlsbad, CA). The Illumina® DNA Prep kit was used to prepare indexed paired end libraries of genomic DNA. Sequencing libraries were normalized to 4 nM, pooled and denatured with 0.2 N sodium hydroxide. Libraries were sequenced on the Illumina MiSeq instrument (Illumina, San Diego, CA, USA). Paired-end fastq reads were assembled using Genome Detective 1.132 (https://www.genomedetective.com) and the Coronavirus Typing Tool[3]. The initial assembly obtained from Genome Detective was polished by aligning mapped reads to the references and filtering out low-quality mutations using bcftools 1.7-2 mpileup method. Mutations were confirmed visually with bam files using Geneious software (Biomatters Ltd, New Zealand).

Clade and Lineage classification and phylogenetic analysis

To assign the sequenced samples to their lineage and clade, we used the dynamic lineage classification method proposed by Rambault et al. via the Phylogenetic Assignment of named Global Outbreak LINeages (PANGOLIN) software suite (https://github.com/hCoV-2019/pangolin)[4], and Nextclade[5], respectively. We confirmed clade classification by analyzing our sequenced genomes against a global reference dataset using a custom pipeline based on a local version of NextStrain (https://github.com/nextstrain/ncov)[5]. The pipeline contains several python scripts that manage the analysis workflow. It performs alignment of genomes in MAFFT[6], phylogenetic tree inference in IQ-Tree V1.6.9[7], tree dating and ancestral state construction and annotation (https://github.com/nextstrain/ncov). The phylogenetic trees were visualized using ggplot and ggtree[8,9].

Expression and purification of SARS-CoV-2 antigens

SARS-CoV-2 spike and receptor binding domain + subdomain 1 proteins were expressed in Human Embryonic Kidney (HEK) 293F suspension cells by transfecting the cells with SARS CoV-2 plasmid DNA. After incubating for six days at 37 °C, 70% humidity and 10% CO_2, proteins were first purified using a nickel resin followed by size-exclusion chromatography. Relevant fractions were collected and frozen at -80 °C until use.

Enzyme-linked Immunosorbent Assay (ELISA)

Two μg/ml of full spike or receptor binding domain + subdomain 1 proteins were used to coat 96-well, high-binding plates and incubated overnight at 4 °C. The plates were blocked in blocking buffer consisting of 5% skimmed milk powder, 0.05% Tween 20, 1X PBS. Plasma

samples were diluted to 1:100 starting dilution in blocking buffer and added to the plates. Secondary antibody was diluted to 1:3000 in blocking buffer and added to the plates followed by 3,3',5,5'-tetramethylbenzidine (TMB) substrate (Thermofisher Scientific). Upon stopping the reaction with 1M H_2SO_4, absorbance was measured at a 450nm wavelength. In all instances, mAbs CR3022 or palivizumab were used as positive and negative controls, respectively.

SARS-CoV-2 pseudovirus based neutralization assay

SARS-CoV-2 pseudotyped lentiviruses were prepared by co-transfecting the HEK 293T cell line with either the SARS-CoV-2 614G spike (D614G), SARS-CoV2 501Y.V2-receptor binding domain spike (K417N, E484K and N501Y, D614G) or SARS-CoV-2 501Y.V2 spike (L18F, D80A, D215G, K417N, E484K, N501Y, D614G, A701V, 242-244 del) and the SARS-CoV-2 501Y.V3 spike (L18F, T20N, P26S, D138Y, R190S, K417T, E484K, N501Y, D614G, H655Y, T1027I, V1176F) plasmids in conjunction with a firefly luciferase encoding lentivirus backbone plasmid and a murine leukemia virus backbone plasmid. The parental plasmids were kindly provided by Drs Elise Landais and Devin Sok (IAVI). For the neutralization assay, heat-inactivated plasma samples from COVID-19 convalescent donors were incubated with the SARS-CoV-2 pseudotyped virus for 1 hour at 37°C, 5% CO_2. Subsequently, $1x10^4$ HEK293T cells engineered to over-express ACE-2, kindly provided by Dr Michael Farzan (Scripps Research), were added and incubated at 37°C, 5% $C0_2$ for 72 hours upon which the luminescence of the luciferase gene was measured. CB6, CA1, CR3022 and palivizumab were used as controls.

Figures and Tables

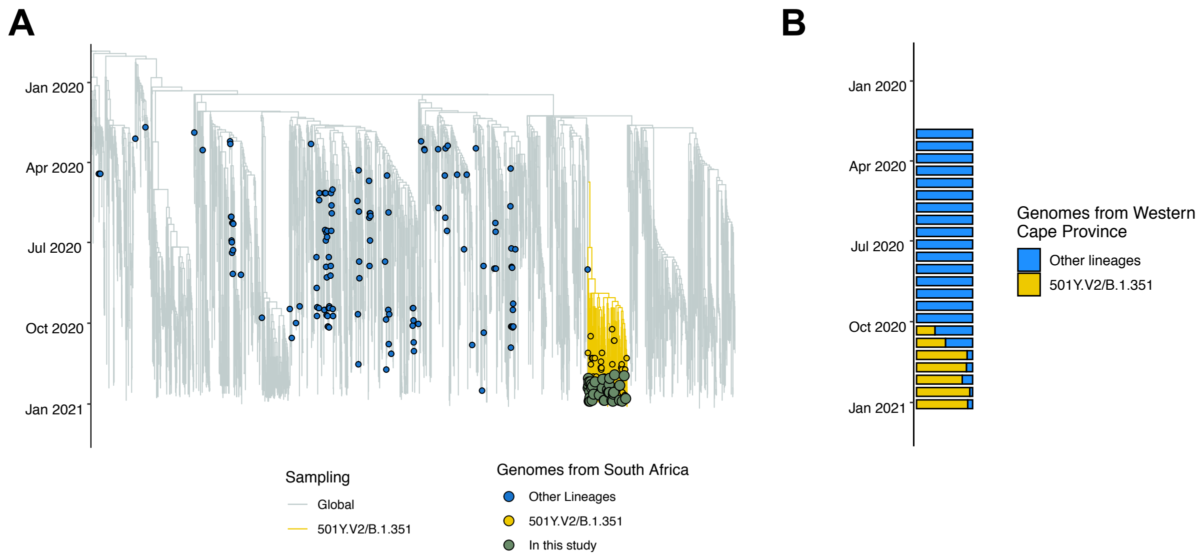

Figure S1. SARS-CoV-2 genome sequencing confirms dominance of infection by the 501Y.V2 variant in Western Cape, South Africa. Genomic sequencing was conducted in the Western Cape Province of South Africa (the same province where our GSH cohort is based) on an approximately weekly basis from the second week of March 2020 to the second week of January 2020. Swabs from 28 individuals from the GSH cohort were sequenced to confirm the infecting variant. (**A**) A time-resolved maximum clade credibility phylogeny of 2621 SARS-CoV-2 sequences, 209 of which are from South Africa and denoted with tip points. The 501Y.V2 (B.1.351) cluster is highlighted in yellow, and the genomes from this study are shown in green, all falling in that cluster. One genome, although assigned to the 501Y.V2/B.1.351 lineage by mutations, was excluded from the phylogenetic tree because of low coverage. (**B**) The bar plot shows the progressive proportion of the 501Y.V2/B.1.351 lineage in the Western Cape.

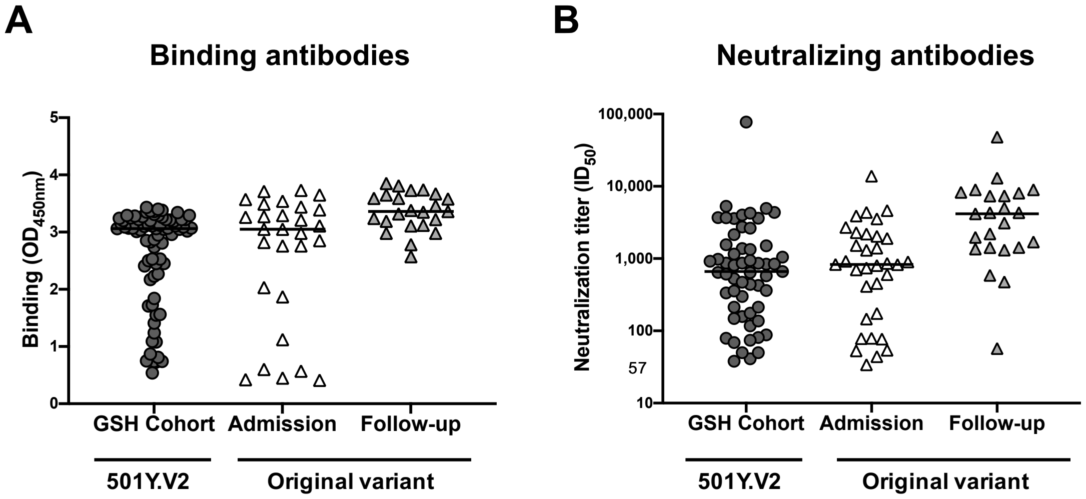

GMT	2,4 (2,2-2,7)	2,1 (1,5-2,9)	3,3 (3,2-3,5)
Fold change		0,9	1,4

GMT	618 (413-927)	649 (372-1133)	2995 (1657-5415)
Fold change		1,1	4,8

Figure S2. 501Y.V2 elicits robust binding and neutralizing antibody responses. Plasma samples from hospitalized individuals infected with either the 501Y.V2 variant (n=89) or the original variant (n=62) were tested for (**A**) Binding (OD_{450nm}) to sequence-matched SARS-CoV-2 spike antigen and (**B**) neutralizing activity (ID_{50}) against sequence-matched SARS-CoV-2 pseudovirus. Binding was assessed at a single dilution of 1:100, and neutralization titers from a starting dilution of 1:20. For both binding and neutralization, only individuals with binding or neutralizing responses are shown. For the 501Y.V2 cohort, binding data are shown for 75 patients, and neutralization data for 57. For the original variant cohort, 28 samples were measured at admission and 23 at follow-up for binding responses; 33 at admission and 23 at follow-up for neutralization assays. Samples were scored as positive when binding was greater than an $OD_{450nm}>0.4$ and the threshold of detection for the neutralization assay is $ID_{50}>20$. All experiments were performed in duplicate and the average value is shown. The median for each dataset is indicated. Geometric mean titers (GMT) (95% confidence interval) were calculated for each group and the fold change relative to the titers of the Groote Schuur Cohort are shown.

A

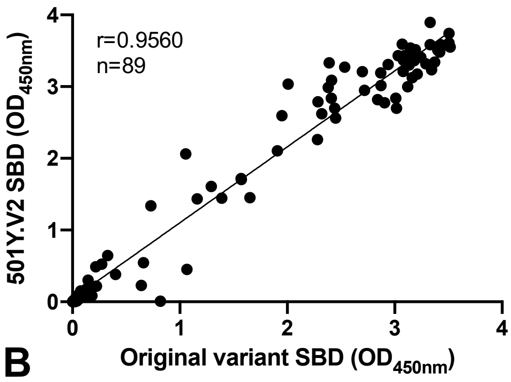

B

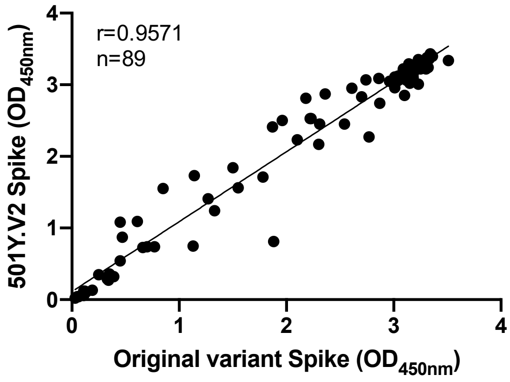

C

Area under the curve - Spike antigen		
501Y.V2	Original	Fold Change
615	184	3,3
1,213	1,590	0,8
1,323	310	4,3
1,492	424	3,5
1,721	1,360	1,3
2,442	5,865	0,4
2,481	1,747	1,4
2,747	1,259	2,2
2,777	3,792	0,7
2,823	5,693	0,5
3,037	2,697	1,1
3,193	3,229	1,0
5,317	3,656	1,5
5,576	2,682	2,1
6,148	5,230	1,2
8,431	2,115	4,0
8,589	870	9,9
11,251	3,391	3,3
12,148	9,842	1,2
13,708	8,910	1,5
18,395	9,917	1,9
18,719	12,760	1,5
20,126	14,575	1,4
22,709	12,659	1,8
22,861	15,657	1,5
23,583	12,181	1,9
23,982	19,815	1,2
24,077	22,203	1,1
24,716	5,850	4,2
24,917	14,803	1,7
25,204	12,453	2,0
25,768	17,997	1,4
26,252	20,510	1,3
26,420	17,218	1,5
26,533	17,107	1,6
26,623	21,979	1,2
29,383	17,515	1,7
33,260	24,270	1,4
34,30	1960	17,5
34,351	20,615	1,7
34,545	21,599	1,6
35,387	24,960	1,4
36,015	25,000	1,4
36,738	22,221	1,7
37,428	28,235	1,3
57,234	29,494	1,9

Area Under the Curve
100-10,000
10,001-20,000
>20,000

Figure S3. Plasma binding antibodies in 501Y.V2 infected individuals are cross-reactive. Plasma samples from 89 GSH patients were tested for binding to (**A**) receptor binding domain + S1 domain (SBD) (original variant and 501Y.V2) and (**B**) full spike (original variant and 501Y.V2). Binding in (A) and (B) was assessed at a single plasma dilution of 1:100. (**C**) Plasma samples from a subset of n=46 individuals were titrated out in full against the 501Y.V2 spike (column 1) and the spike from the original variant (column 2). Data are plotted as area under the curve, ranked by titers against 501Y.V2, and colored according to magnitude. The fold differences between binding to each of the two lineages is shown in column 3, with grey shading denoting fold changes ≥2. All experiments were performed in duplicate and the average value is shown.

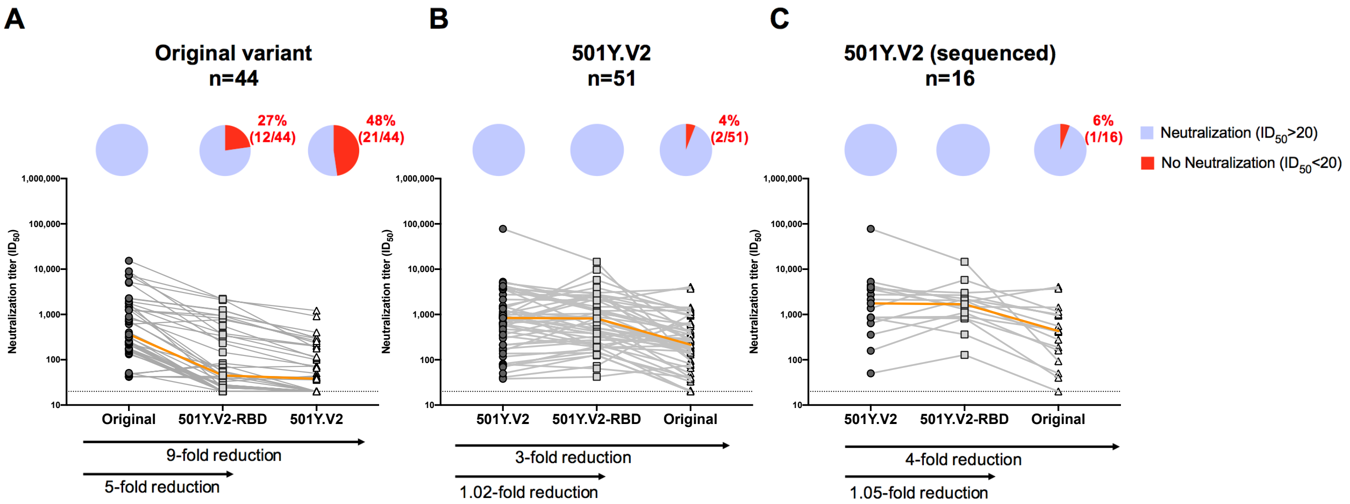

Figure S4. Antibody cross-reactivity against the full 501Y.V2, 501Y.V2-RBD and the original variant in plasma from patients infected with 501Y.V2. (**A**) Plasma samples from patients infected with the original variant and (**B-C**) 501Y.V2-infected GSH cohort samples were compared for their neutralization cross-reactivity against 501Y.V2, 501Y.V2-RBD and D614G (n=51). In (C), the analysis was limited to those samples where sequencing confirmed infection by 501Y.V2 (n=16). The construct that contains only the three receptor binding domain changes in 501Y.V2 (K417N, E484K, N501Y) showed similar titers to the full 501Y.V2 (GMT:669 (462-969) compared to 501Y.V2: GMT:686 (448-1052), for this subset of n=51). The orange line indicates the slope between the median neutralization potency of the samples tested. Two plasma samples showed the opposite trend, with higher neutralization of the original variant (>5 fold-higher), compared to 501Y.V2. We do not have sequencing data for these samples but it is likely that these individuals were infected with the original variant and not 501Y.V2. In the pie charts, purple indicates the proportion of samples with neutralization activity and red the proportion of samples with no detectable neutralization activity. The threshold of detection for the neutralization assay is ID_{50}>20. All experiments were performed in duplicate and the average value is shown. Data for the original virus plasma was taken from *Wibmer et al., 2021, Nature Medicine*.

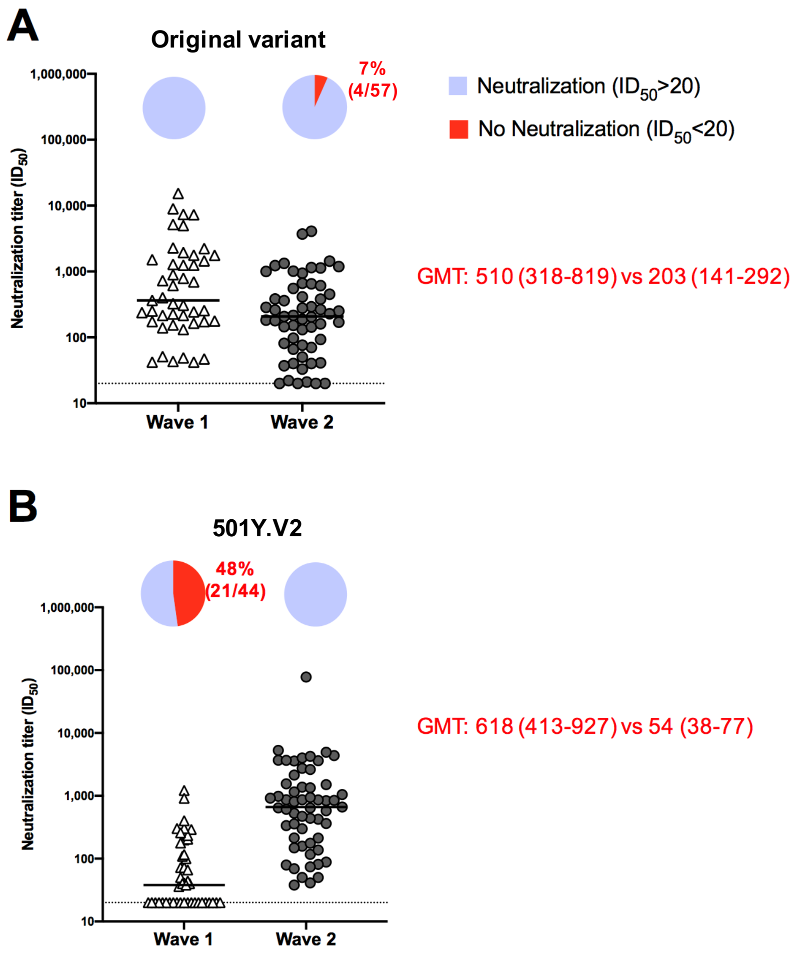

Figure S5. Plasma elicited by 501Y.V2 infection (Wave 2) is more cross-reactive than plasma from the original virus infection (Wave 1). Plasma from patients infected with 501Y.V2 or the original variant were tested against (A) the original virus or (B) the 501Y.V2 variant. We compared titers from wave 1, where infection was by the original variant (May to September 2020) and the wave 2 Groote Schuur Hospital plasma (501Y.V2 infection, December 2020 to January 2021) against both the original variant (Figure S3A) and 501Y.V2 (Figure S3B). The original variant was neutralized by both wave 1 and wave 2 sera (GMT: 510 and 203, respectively). However, the 501Y.V2 variant was neutralized 11-fold better by the wave 2 sera (GMT: 618) than wave 1 sera (GMT: 54) which was obtained from both hospitalized and non-hospitalized individuals (Figure S3A, B). 95% confidence intervals for each geometric mean are shown in brackets. In the pie charts, purple indicates the proportion of samples with neutralization activity and red the proportion of samples with no detectable neutralization activity. The threshold of detection for the neutralization assay is $ID_{50}>20$. All experiments were performed in duplicate and the average value is shown. Data for the original virus plasma was taken from *Wibmer et al., 2021, Nature Medicine*.

Table S1: Summary of patient information and of samples tested in binding and neutralization assays summary

Sample ID	Sex	Age	PCR-positive Test Date	Date blood sample collected	Days since PCR-positive test	Swabs tested in viral sequencing	Binding (OD450nm) Original Variant SBD (1:100)	Binding (OD450nm) 501Y.V2 SBD (1:100)	Binding (OD450nm) Original Variant Spike (1:100)	Binding (OD450nm) 501Y.V2 Spike (1:100)	Binding (OD450nm) Binding Spike titration (AUC)	Neutralization (ID50) Original Variant	Neutralization (ID50) 501Y.V2-RBD	Neutralization (ID50) 501Y.V2	Neutralization (ID50) 501Y.V3
SA-01-0001	Female	30	2020/12/18	2020/12/30	12										
SA-01-0002	Male	51	2020/12/16	2020/12/30	14										
SA-01-0003	Male	58	2020/12/27	2020/12/30	3										
SA-01-0004	Male	31	2020/12/25	2020/12/30	5										
SA-01-0005	Male	50	2020/12/13	2020/12/30	17										
SA-01-0006	Female	48	2020/12/09	2020/12/30	21										
SA-01-0008	Male	75	2020/12/23	2020/12/30	7										
SA-01-0009	Male	51	2020/12/19	2020/12/31	12										
SA-01-0010	Female	47	2020/12/21	2020/12/31	10										
SA-01-0012	Female	40	2020/12/21	2020/12/31	10										
SA-01-0013	Female	52	2020/12/21	2020/12/31	10										
SA-01-0014	Male	64	2020/12/28	2020/12/31	3										
SA-01-0015	Male		2020/12/23	2020/12/31	8										
SA-01-0016	Female	47	2020/12/30	2021/01/05	6										
SA-01-0017	Female	68	2020/12/24	2021/01/05	12										
SA-01-0018	Male	63	2020/12/07	2021/01/05	29										
SA-01-0019	Male	26	2021/01/02	2021/01/05	3										
SA-01-0020	Male	59	2020/12/28	2021/01/05	8										
SA-01-0021	Male	56	2020/12/28	2021/01/05	8										
SA-01-0022	Male	48	2021/01/03	2021/01/05	2										
SA-01-0023	Male	49	2020/12/30	2021/01/05	6										
SA-01-0024	Female	59	2020/12/16	2021/01/05	20										
SA-01-0025	Female	57	2020/12/22	2021/01/05	14										
SA-01-0026	Male	49	2020/12/26	2021/01/06	11										
SA-01-0027	Male	49	2020/12/28	2021/01/06	9										
SA-01-0028	Male	68	2020/12/17	2021/01/06	20										
SA-01-0029	Male	54	2020/12/26	2021/01/06	11										
SA-01-0030	Male	40	2021/01/04	2021/01/06	2										
SA-01-0031	Female	61	2021/01/04	2021/01/06	2										
SA-01-0032	Female	65	2020/12/23	2021/01/06	14										
SA-01-0033	Male	55	2021/01/03	2021/01/06	3										
SA-01-0035	Male	38	2020/12/21	2021/01/06	16										
SA-01-0036	Male	44	2021/01/04	2021/01/06	2										
SA-01-0037	Male	60	2021/01/02	2021/01/07	5										
SA-01-0038	Male	49	2020/12/25	2021/01/07	13										
SA-01-0039	Male	38	2021/01/01	2021/01/07	6										
SA-01-0040	Male	50	2021/01/05	2021/01/07	2										
SA-01-0041	Male	79	2021/01/04	2021/01/07	3										
SA-01-0042	Female	43	2020/12/09	2021/01/07	29										
SA-01-0044	Female	51	2021/01/03	2021/01/07	4										
SA-01-0045	Female	66	2021/01/03	2021/01/07	4										
SA-01-0047	Male	41	2021/01/04	2021/01/07	3										
SA-01-0048	Male	71	2021/01/05	2021/01/07	2										
SA-01-0050	Female	53	2020/12/31	2021/01/08	8										
SA-01-0051	Female	68	2021/01/02	2021/01/08	6										
SA-01-0052	Female	67	2021/01/03	2021/01/08	5										
SA-01-0053		53	2021/01/01	2021/01/08	7										
SA-01-0054	Female	56	2021/01/01	2021/01/08	7										
SA-01-0055	Female	56	2020/12/19	2021/01/08	20										
SA-01-0056	Male	71	2021/01/02	2021/01/08	6										
SA-01-0057	Female	44	2021/01/05	2021/01/08	3										
SA-01-0058	Male	53	2020/12/30	2021/01/08	9										
SA-01-0059	Male	25	2020/12/28	2021/01/08	11										
SA-01-0060	Male	50	2021/01/05	2021/01/08	3										
SA-01-0061	Male	57	2020/12/18	2021/01/12	25										
SA-01-0062		51	2021/01/07	2021/01/12	5										
SA-01-0063	Male	45	2020/12/23	2021/01/12	20										
SA-01-0064	Female	59	2021/01/04	2021/01/12	8										
SA-01-0066	Male	64	2021/01/10	2021/01/12	2										
SA-01-0067	Male	45	2021/01/07	2021/01/12	5										
SA-01-0068	Male	34	2020/12/23	2021/01/12	20										
SA-01-0069	Male	49	2021/01/09	2021/01/12	3										
SA-01-0070	Female	71	2021/01/04	2021/01/12	8										
SA-01-0071	Female	40	2021/01/11	2021/01/12	1										
SA-01-0072	Female	52	2020/12/30	2021/01/12	13										
SA-01-0073	Male	83	2021/01/13	2021/01/13	0										
SA-01-0075	Male	76	2021/01/03	2021/01/13	10										
SA-01-0076	Male	33	2021/01/08	2021/01/13	5										
SA-01-0077	Female		2021/01/05	2021/01/13	8										
SA-01-0078	Female	73	2021/01/05	2021/01/13	8										
SA-01-0079	Female	53	2021/01/09	2021/01/13	4										
SA-01-0080	Female	64	2021/01/11	2021/01/13	2										
SA-01-0081	Female	67	2020/12/30	2021/01/13	14										
SA-01-0082	Male	58	2021/01/11	2021/01/13	2										
SA-01-0084	Female	60	2021/01/12	2021/01/14	2										
SA-01-0085	Female	77	2021/01/09	2021/01/14	5										
SA-01-0086	Female	38	2021/01/11	2021/01/14	3										
SA-01-0087	Female	40	2020/12/24	2021/01/14	21										
SA-01-0088	Female	73	2020/12/30	2021/01/14	15										
SA-01-0089	Female	59	2021/01/10	2021/01/14	4										
SA-01-0091	Male	60	2021/01/11	2021/01/14	3										
SA-01-0092	Female	68	2021/01/04	2021/01/15	11										
SA-01-0094	Female	55	2021/01/11	2021/01/15	4										
SA-01-0095	Male	42	2021/01/09	2021/01/15	6										
SA-01-0096	Male	66	2021/01/12	2021/01/15	3										
SA-01-0098	Male	76	2021/01/06	2021/01/15	9										
SA-01-0099	Female	65	2021/01/05	2021/01/15	10										
SA-01-0100	Female	56	2020/12/25	2021/01/15	21										
SA-01-0101	Female	71	2021/01/06	2021/01/15	9										

KEY	
(black)	Positive
(grey)	Negative
(white)	Not tested

Acknowledgements

We thank the participants in the GSH cohort and staff who helped in the collection of samples. Mieke van der Mescht, Zelda van der Walt, Talita de Villiers, Fareed Abdullah, Paul Rheeder, Albertus Malan, Simon Spoor and Wesley van Hougenhouck-Tulleken are thanked for contributing to patient management, sample collection and processing, and data management for the Pretoria COVID study. We thank Richard Baguma, Roanne Keeton and Marius Tincho for sample processing and shipping. We thank Stephen Korsman and Tamryn Smith for assistance with sample identification and access. We thank Drs Nicole Doria-Rose, David Montefiori, Elise Landais and Michael Farzan for reagents and assistance in establishing the SARS-CoV-2 pseudotyped neutralization assay and enabling equivalency and proficiency testing. We thank Drs Devin Sok, Elise Landais, Dennis Burton, Nicole Doria-Rose and Peter Kwong for SARS-CoV-2 directed mAbs. We are grateful to Jason McLellan for the WT Hexapro Spike construct. We thank Anne von Gottberg, Cheryl Cohen and Susan Meiring for samples in the previously published comparator group. We thank the informal 501Y.V2 consortium of South African scientists, chaired by Drs Willem Hanekom and Tulio de Oliveira for suggestions and discussion of data, and Drs Valerie Mizrahi and Robert Wilkinson for enabling this collaboration. We also thank all NGS-SA laboratories in South Africa that were responsible for producing the SARS-CoV-2 genomes that enabled the rapid dissemination of SARS-CoV-2 sequences.

Author Contributions

T.M-G. performed and designed experiments, analyzed data and wrote the manuscript. M.M performed neutralization experiments and analyzed data. Z.M and F.A performed ELISA binding experiments. D.M, B.O and B.E.L made molecular constructs. P.K performed neutralization assays. H.T, T.dO, A.I, D.D, L.T and C.W sequenced the viral genomes. L.R.C, M.M, S.S and G.M aided in sample collection, preparation and cohort organization. C.K.W and J.N.B designed molecular constructs and provided intellectual input to the study design. J.N.B, C.W and T.d.O identified the 501Y.V2 lineage. T.R, M.B and V.U provided plasma samples for the study. W.A.B and N.N provided samples for the study and clinical data on the participants. L.M aided in the establishment of the pseudovirus assay and intellectual input to the study design. P.L.M conceptualized the study, designed experiments and wrote the manuscript.

Conflicts of Interest

None of the authors declare any conflicts of interest

References

1. Wibmer, C.K., F. Ayres, T. Hermanus, M. Madzivhandila, P. Kgagudi, B. Oosthuysen, et al., SARS-CoV-2 501Y.V2 escapes neutralization by South African COVID-19 donor plasma. Nature Medicine, 2021.

2. Pillay, S., J. Giandhari, H. Tegally, E. Wilkinson, B. Chimukangara, R. Lessells, et al., Whole genome sequencing of SARS-CoV-2: adapting Illumina protocols for quick and accurate outbreak investigation during a pandemic. Genes, 2020. 11(8): p. 949.

3. Cleemput, S., W. Dumon, V. Fonseca, W. Abdool Karim, M. Giovanetti, L.C. Alcantara, et al., Genome Detective Coronavirus Typing Tool for rapid identification and characterization of novel coronavirus genomes. Bioinformatics, 2020. 36(11): p. 3552-3555.

4. Rambaut, A., E.C. Holmes, Á. O'Toole, V. Hill, J.T. McCrone, C. Ruis, et al., A dynamic nomenclature proposal for SARS-CoV-2 lineages to assist genomic epidemiology. Nature microbiology, 2020. 5(11): p. 1403-1407.

5. Hadfield, J., C. Megill, S.M. Bell, J. Huddleston, B. Potter, C. Callender, et al., Nextstrain: real-time tracking of pathogen evolution. Bioinformatics, 2018. 34(23): p. 4121-4123.

6. Katoh, K. and D.M. Standley, MAFFT multiple sequence alignment software version 7: improvements in performance and usability. Molecular biology and evolution, 2013. 30(4): p. 772-780.

7. Nguyen, L.-T., H.A. Schmidt, A. Von Haeseler, and B.Q. Minh, IQ-TREE: a fast and effective stochastic algorithm for estimating maximum-likelihood phylogenies. Molecular biology and evolution, 2015. 32(1): p. 268-274.

8. Yu, G., D.K. Smith, H. Zhu, Y. Guan, and T.T.Y. Lam, ggtree: an R package for visualization and annotation of phylogenetic trees with their covariates and other associated data. Methods in Ecology and Evolution, 2017. 8(1): p. 28-36.

9. Wickham, H., ggplot2: Elegant Graphics for Data Analysis. Springer-Verlag: New York, NY, USA., 2016.
